# Supplementary figures and images for: Altered light induced EGR1 expression in the SCN of PACAP deficient mice
Source: PLoS One. 2020 May 7;15(5):e0232748. doi: 10.1371/journal.pone.0232748 (PMC7205239; doi:10.1371/journal.pone.0232748)

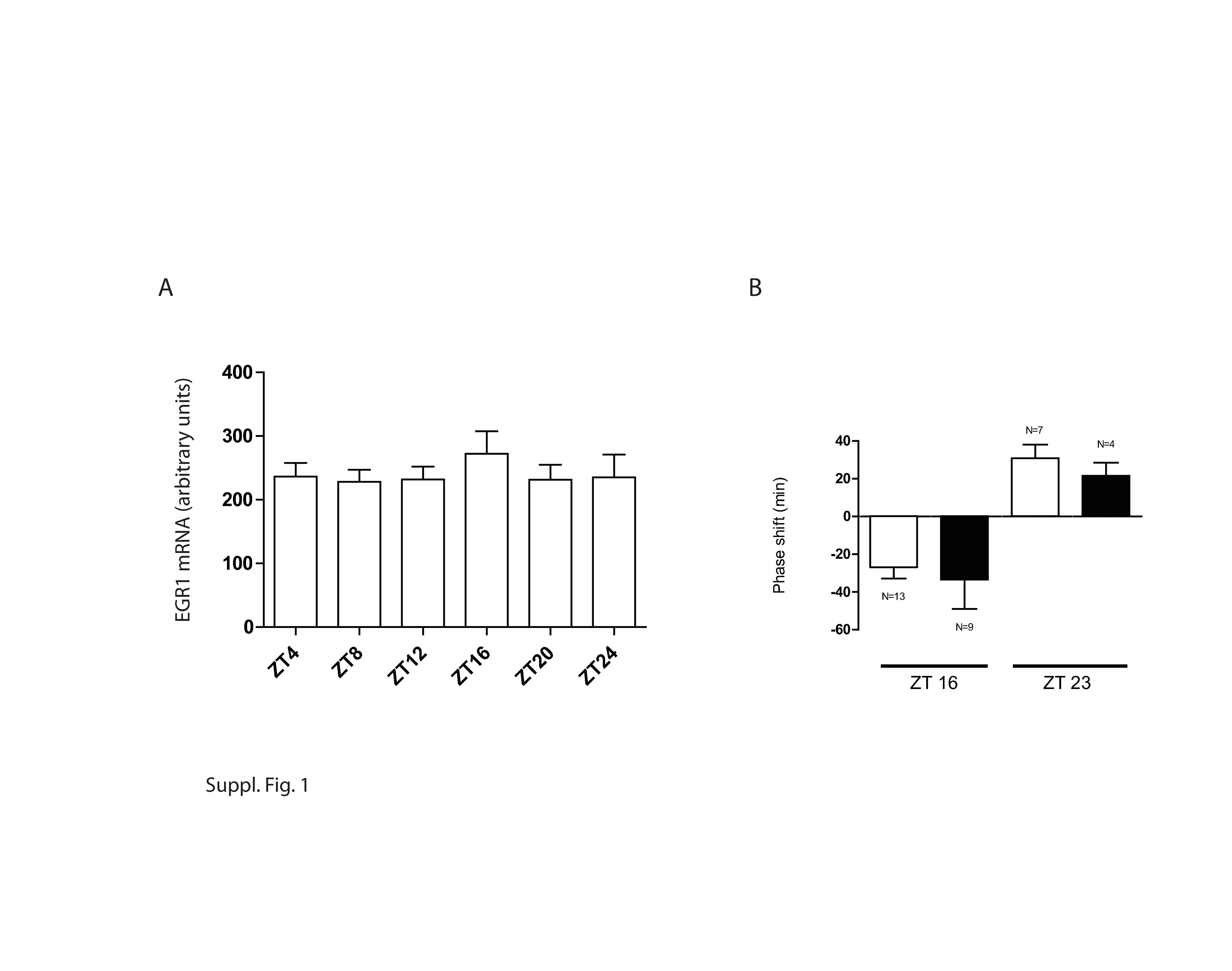

Supplement: S1 Fig — (A) Egr1 mRNA expression during a 24 h LD cycle. Values were analysed using one-anova followed by Bonferroni’s Multiple Comparison Test. P < 0.05 was considered statistically significant. Values are given as mean ± SEM (n = 8 in each group, 4 males and 4 females). (B) Light induced phase shifts in wild type (EGR1+/+, black) and EGR1 deficient (EGR1-/-, white) mice during early (ZT16) and late subjective (ZT23) night to a 30 minutes light stimulation (10 lux). Values are given as mean ± SEM (n = 7–13 in each group). NS: not significant using Mann-Whitney U test. (TIF) [file pone.0232748.s001.tif]
