# Supplementary material for: Altered light induced EGR1 expression in the SCN of PACAP deficient mice
Source: PLoS One. 2020 May 7;15(5):e0232748. doi: 10.1371/journal.pone.0232748 (PMC7205239; doi:10.1371/journal.pone.0232748)
Supplement: S1 Material — (DOCX) [file pone.0232748.s002.docx]

**Supplementary material and methods**

**Egr1 mRNA expression after light stimulation at night and during a 24 h LD cycle**

Animals used for light experiments killed after 30 and 120 min

32 PACAP wild type (+/+) and 32 PACAP deficient (-/-) (16 males and 16 females of each genotype, age 8-12 weeks when included in the study) mice were bred from heterozygote animals. All animals included in the study were maintained in a 12:12 h light/dark (LD) cycle (light on at 6 a.m. = Zeitgeber (ZT) 0, lights off at 6 p.m. = ZT12) and housed in individual cages with food (Altromin 1324; Altromin Spezialfutter, Germany) and water ad libitum unless otherwise stated. For light-stimulation experiments, animals received a 30 min pulse of white light (>300 lux) at ZT16. For in situ hybridization, animals (n = 6-8 in each group, equal number of sexes) were decapitated (in dim red light, <3 lux), 30 min and 120 min after the initiation of light stimulation. The same number of time match darkness controls were decapitated in dim red light, <3 lux.

Another group of PACAP wild type (+/+) (48 in total, 24 males and 24 females) kept in in a 12:12 h LD cycle (light on at 6 a.m. = Zeitgeber (ZT) 0, lights off at 6 p.m. = ZT12) were killed by decapitation every four h during the 24 h cycle (ZT4, 8, 12, 16, 20, 24). Animals were treated according to the principles of Laboratory Animal Care (Law on Animal Experiments in Denmark, publication 382, June 10, 1987) and under Danish Veterinary Authorities (Dyreforsoegstilsynet) license no. 2008/561-1445. The animal research ethics committee (Dyreforsoegstilsynet) granted a formal waiver of ethics approval license no: 2017-15-0201-01364 to Jens Hannibal and thereby approved the study.

Animals used for running wheel experiments

The EGR1 colony of mice were founded from six (three of each gender) heterozygous animals purchased from Charles Rivers Laboratories (Sulzfeld, Germany)[1]. All animals (equal number of each gender and genotype) included in the study were maintained in a 12:12-h LD cycle and housed in individual cages with food (Altromin 1324; Altromin Spezialfutter, Germany) and water ad libitum unless otherwise stated. Animals were treated according to the principles of Laboratory Animal Care (Law on Animal Experiments in Denmark, publication 382, June 10, 1987) and under Danish Veterinary Authorities (Dyreforsoegstilsynet) license no. 2017-15-0201-01364 to Jens Hannibal and the study was thereby approved.

#### Light-induced phase shift

### Using wheel-running activity, light induced phase shift of the circadian rhythm was determined using the Aschoff type II regime as described previously [1, 2]. All animals were light stimulated for 30 min at 10 lux in their home-cages in separate experiments at ZT16 and ZT23, respectively, after which the lights were turned off for the next 10–14 days followed by 14 days of re-entrainment in LD before the next light pulse experiment.

### Data Analysis

Data obtained from the Mini Mitter, Running Wheel activity system and the ER-4000 energizer receiver system were analyzed in ClockLab (ActiMetric Software, Coulbourn Instruments) running under Matlab (R2012a, Windows7, 64-bit; MathWorks, Natick, MA) environment. Light induced phase shifts were determined using ClockLab applied on the actograms. The light induced phase shift was determined using the difference in phase from a regression line drawn by Clocklab through the activity onset of the entrained (LD) onset at least 7 days immediately before the day of stimulation, and a regression line drawn from the onset from two-three days after light stimulation (to avoid any mislead due to transients) of the free running activity onsets (DD)[1].

EGR1 expression during 24 LD cycle

Animals from ZT4, 8 and 12 were killed during white light conditions whereas animals from ZT16, 18 and 24 were killed by decapitation in dim red light. All animals had their brain removed and frozen until cutting in a freezing microtome.

#### Measurements of wheel-running activity rhythms

EGR1+/+ and EGR1-/- mice were transferred to individual cages equipped with a running wheel (diameter: 23 cm, 4 magnets/wheel) in ventilated chambers with controlled white lighting (300 lux)[1]. Wheel-running activity was monitored by an online personal computer connected via a magnetic switch to the Running Wheel Activity System (consisting of QA-4 activity input modules, DP-24 dataports, and Vital View data acquisition system version 4.1; Mini Mitter, Sunriver, OR). Wheel revolutions were collected continuously in 6-min bins. Animals were entrained to a LD cycle [lights on at 7:00 AM designated ZT = 0, off at 7:00 PM = ZT12] for at least 14 days before start of experiments[1].

*Fluorescence in situ hybridization (FISH) using digoxigenin labelled antisense RNA probes*

In situ hybridization for EGR1 mRNA from animals killed 30 min and 120 min after initiations of the light pulse was performed using digoxigenin labeled probes [3]. Briefly, brain sections were cut on a cryostat in 12-μm-thick coronal sections through the SCN in three series of five slides with 3-4 sections on each slide, as described in the main text. From each animal, one gelatin-coated slide from each series representing the rostral, mid, and caudal part of the SCN, respectively, was hybridized with the Dig-labeled *Egr1* antisense probe (diluted 1:1600, details on probe sequence can be found in the main text). After hybridization and washing, slides were incubated with a goat anti-digoxigenin peroxidase conjugated antibody (diluted 1:50) followed by washing and incubation in Alexa Fluor 488-conjugated tyramide (Molecular Probes, diluted 1:100). After washing, slides were mounted in glycerol DAPI solution [4].

Measuring EGR1 mRNA expression

Images used for semi-quantitative FISH were obtained using an iMIC confocal microscope (Till Photonics, FEI, Germany)[4]. The iMIC used an Andromeda spinning disk system (FEI, Germany) and a Hamamatsu 16-bit camera (model C10600-10B-H, Hamamatsu Photonic, Japan) for recording. The microscope was equipped with filter settings for detecting DAPI and CY2/Alexa-488 and the following objective: X20, NA = 0.75. All sections used for quantification were measured using an identical protocol with fixed exposure time (10 ms) and laser intensity at 100 %. At each section on the middle of the SCN (region of most light responding neurons), images were obtained where signal intensity was highest as evaluated by the highest level of grey values (16-bit grey scale values are from 0 to 65,000) (Fig. 2).

Quantitative immunohistochemistry was performed as previously described [4] using Fiji software (version 1.47q, NIH, USA, RRID:SCR_003070) after calibration on a 16-bit grey tone images obtained using identical settings in the microscope (exposure time 10 ms,100 % laser intensity) for optical density (O.D.) as described (<https://imagej.nih.gov/ij/docs/examples/calibration/>).

All images were adjusted for brightness and contrast in Fiji and mounted into plates in Adobe Illustrator CS5 (Adobe, RRID:SCR_010279).

## Statistics

Statistics were performed using GraphPad Prism version 5.0. For comparison one-way Anova followed by Bonferroni’s Multiple Comparison Test was used. P < 0.05 was considered statistically significant. For comparison of two independent groups, the Mann-Whitney U-test was used.

**Results**

EGR1 gene expression during a 24 LD cycle

Quantitative ISH demonstrated that EGR1 mRNA expression in the mouse SCN did not change significantly during a 24 h LD cycle (Suppl. Fig. 1A). A small but insignificant increase in EGR1 mRNA was found in the middle of the subjective night (Suppl. Fig. 1A) corresponding to the cells seen in Fig. 2, upper panel.

Light induced phase shift in EGR1 deficient mice at low (10 lux) light intensities

EGR1 wild type and EGR1 deficient mice exposed to a 30 min light pulse at ZT16 or/and at ZT23 demonstrated a light induced phase delay and phase advance, respectively (Suppl. Fig.1 B). No difference was found between the two genotypes.
